# Prey selection by leopards (*Panthera pardus fusca*) in the mid‐hill region of Nepal

**DOI:** 10.1002/ece3.10924

**Published:** 2024-02-05

**Authors:** Kedar Baral, Shivish Bhandari, Binaya Adhikari, Ripu M. Kunwar, Hari P. Sharma, Achyut Aryal, Weihong Ji

**Affiliations:** ^1^ School of Natural Science Massey University Auckland New Zealand; ^2^ Ministry of Industry, Tourism, Forest and Environment Pokhara Kaski Nepal; ^3^ Morgan State Univeristy Baltimore Maryland USA; ^4^ University Kentucky Lexington Kentucky USA; ^5^ Florida Atlantic University Boca Raton Florida USA; ^6^ Central Department of Zoology Tribhuvan University Kathmandu Nepal; ^7^ Auckland College of Tertiary Studies/CC Training Academy Takapuna, Auckland New Zealand

**Keywords:** diet, leopard, livestock, scat, wild prey

## Abstract

Information on prey selection and the diet of the leopard (*Panthera pardus fusca*) is essential for leopard conservation. We conducted an investigation into the prey species and the proportion of each species in the leopard's diet in a human‐dominated mid‐hill region of Nepal. The analysis of 96 leopard scats collected between August 2020 and March 2021 revealed that leopards consumed 15 prey species, including small‐ and medium‐sized mammals and livestock. In addition to these prey species, we also found plastic materials, bird feathers, and some unidentified items in the leopard scats. Wild ungulates (such as barking deer, *Muntiacus muntjak* and wild boar, *Sus scrofa*) constituted only 10% of the biomass in the scats, while livestock contributed 27%, and other wild prey contributed 50%. Among all species, domestic goats had the highest relative biomass in the scats, followed by the jungle cat (*Felis chaus*), domestic dog (*Canis familiaris*), and large Indian civet (*Viverra zibetha*). Similarly, the Indian hare (*Lepus nigricollis*) had the highest proportion of relative individuals present in the scat samples, followed by the jungle cat and the large Indian civet. A lower proportion of biomass from wild ungulates in the leopard's diet and a higher dependency of the leopard on domestic prey and other wild prey indicate a shortage of medium‐sized wild prey, such as barking deer and wild boar, in leopard habitats. Therefore, the conservation of wild prey species, especially medium‐sized prey, is crucial for reducing the leopard's dependence on livestock and mitigating human‐leopard conflicts in the future.

## Graphical abstract

We investigated the prey species and the proportion of each species in the leopard diet in a human‐dominated mid‐hill region of Nepal. The analysis of 96 leopard scats collected between August 2020 and March 2021 revealed 15 prey species consumed by leopards, including small‐ and medium‐sized mammals and livestock.

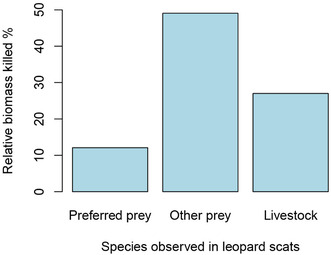

## 1 INTRODUCTION

The leopard (*Panthera pardus fusca*) is one of the mesopredators in forest and grassland ecosystems (Bhandari et al., 2019; Karanth & Sunquist, 1995; Lovari et al., 2015) and is widely distributed in the Indian subcontinent (Baral, Aryal, et al., 2022; Mondal et al., 2011; Thapa, 2011). Leopards are widely distributed in Nepal (Baral et al., 2023), with the mountainous regions of Nepal being one of the most highly suitable habitats for leopards due to their geographical features, diversity of prey species, and vegetation types. Leopards prefer tropical forests both inside and outside the protected areas of Nepal. However, most of the leopard's distribution falls outside the protected areas, where the landscapes are dominated by human and anthropogenic activities (Baral, Aryal, et al., 2022; Bhandari et al., 2019; Thapa, 2011). Leopards are known to adapt to human‐dominated landscapes, such as agricultural lands and patchy forests close to villages (Baral, Aryal, et al., 2022; Bhandari et al., 2019; Mondal et al., 2011). Human population growth and urbanization in Nepal's mountainous regions have reduced the natural habitats of leopards and their prey species. This may have caused leopards to expand their ranges into human‐dominated habitats in search of prey.

Leopards are versatile predators known for their nonselective hunting habits, targeting a wide range of prey species encompassing small‐ to medium‐sized mammals, birds, and livestock (Andheria et al., 2007; Baral, Bhandari, & Adhikari, 2022; Karanth & Sunquist, 1995; Ramesh et al., 2009). Nevertheless, the leopard's morphology and solitary hunting strategy seem to influence its choice of prey, with an apparent preference for species weighing between 10 and 40 kg, with a sweet spot around 25 kg (Baral, Bhandari, & Adhikari, 2022; Hayward et al., 2006). Some studies suggest that, even in areas with abundant large prey, leopards often rely on smaller‐ and medium‐sized game (Bhattarai & Kindlmann, 2012; Thapa, 2011). Karanth and Sunquist (1995) propose that in the absence of large prey, leopards adapt by focusing on medium‐sized quarry. Furthermore, the encroachment of human settlements into leopard habitats raises concerns about shifts in their diet towards domestic animals and unconventional prey, including small mammals and other carnivores (Baral, Bhandari, & Adhikari, 2022; Mondal et al., 2011; Thapa, 2011).

The majority of large Felidae typically favor ungulates as their primary prey. When the abundance of wild ungulates diminishes in their natural habitat due to factors such as population decline or seasonal migrations, domestic species often become more vulnerable (Bhandari et al., 2017; Karanth & Sunquist, 1995; Khorozyan et al., 2015; Ramakrishnan et al., 1999). Instances of livestock depredation tend to rise under these circumstances (Baker et al., 2008; Khorozyan et al., 2015; Zhang et al., 2013). Leopards are known for their frequent attacks on domestic animals, including dogs, goats, and other livestock (Khorozyan et al., 2015; Ramesh et al., 2009; Thapa, 2011). This predation on livestock contributes to negative interactions between humans and leopards, ultimately escalating into human‐leopard conflicts (Bhandari et al., 2019; Kandel et al., 2020; Kumaraguru et al., 2011).

Nepal's mid‐hill region stands as a crucial leopard habitat, but it faces growing challenges (Adhikari, Baral, Bhandari, Kunwar, et al., 2022; Baral, Bhandari, Adhikari, et al., 2022). Deforestation, the illegal hunting of the leopard's prey, and various other forms of human interference have escalated conflicts between people and leopards (Adhikari, Baral, Bhandari, Szydlowski, et al., 2022; Baral et al., 2021; Sharma et al., 2021). This study is of particular significance in human‐dominated landscapes within the mid‐hill regions, especially beyond protected areas (Adhikari, Bhandari, et al., 2022; Baral et al., 2023). Its primary aim is to gain insights into the leopard's prey preferences, shedding light on the intricate relationship between leopards and their dietary ecology, ultimately aiding in conflict mitigation. Furthermore, this research delves into the role of domestic species in the leopard's diet. It seeks to assess the extent of leopard predation on livestock and identify potential causes. Our ultimate objective is to propose measures for the conservation of the leopard's preferred prey species. These efforts aim to alleviate conflicts between humans and leopards while contributing to the conservation of leopards in Nepal's mid‐hill region.

## 2 METHODS

### 2.1 Study site

**FIGURE 1:**
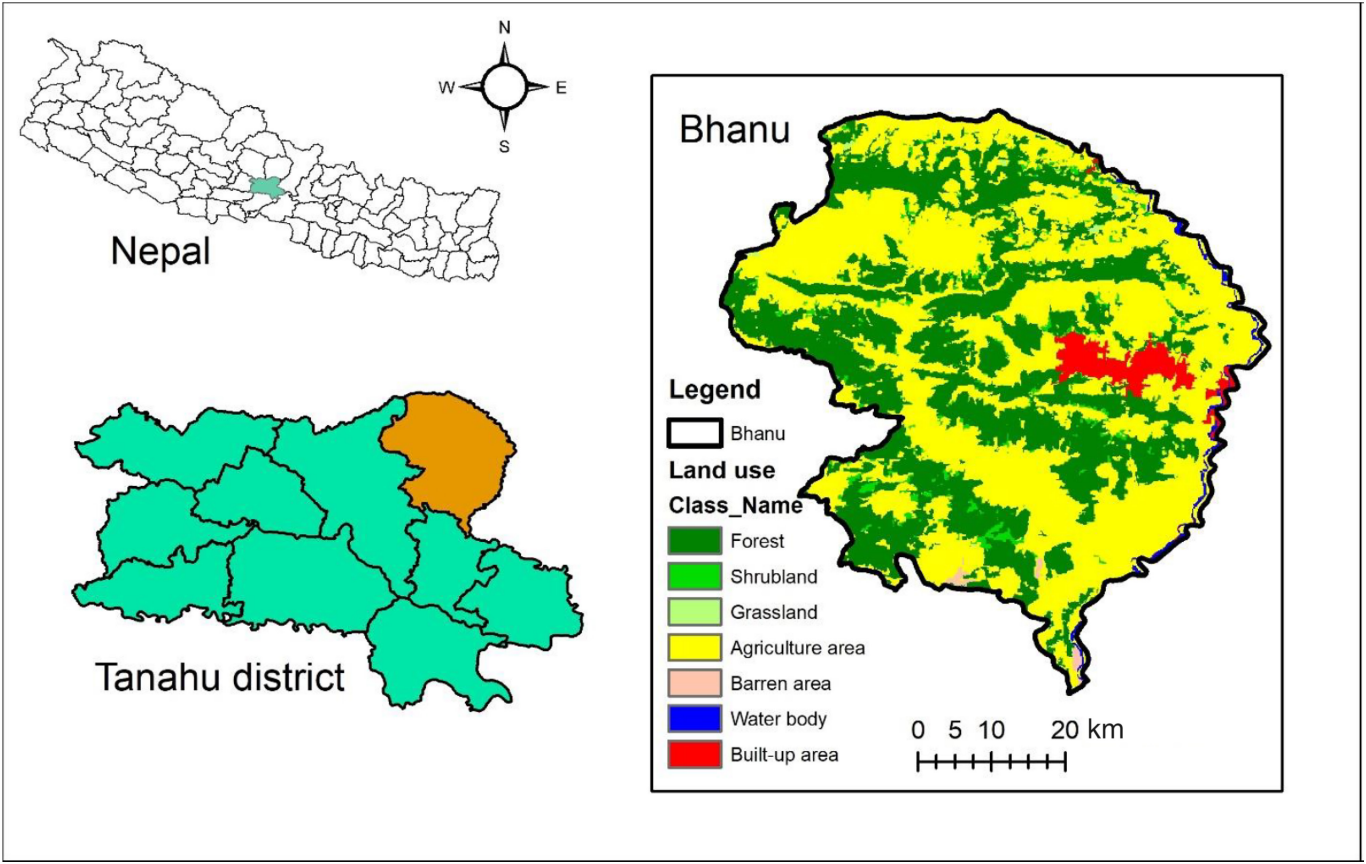
The study site's location in Tanahun, Nepal, with the land‐use and land‐cover map of the study area.

The study site is located in Bhanu municipality (27°26′ to 28°25′ N and 84°57′ to 85°34′ E; altitude 450–800) of Tanahun district in the mid hill region, Nepal (Figure 1). The survey was conducted in the community forests. The community forests were established as a result of a set of policies and institutional innovations that began in the mid‐1970s to involve local communities in forest management and biodiversity conservation, as well as to improve livelihoods (Ghimire & Lamichhane, 2020; Laudari et al., 2020; Uprety et al., 2011). In the study area, forests of different sizes (from 3 to 300 ha) are managed by community forest user groups (Sharma et al., 2021; Uprety et al., 2011). Sal (*Shorea robusta*) dominated forest and riparian forest are the dominant forest types in the study area. The riparian forests are found on sandy soils of the riverbanks. The dominant species is Acacia catechu, which is threatened in the area. Other common plant species include *Bombax ceiba*, *Dalbergia sissoo*, *Sapium insigne*, *Schima wallichii*, *Lagerstroemia parviflora*, *Bauhinia vahlii*, *Desmodiumo ojeinense*, and *Murraya koenigii* (Adhikari et al., 2023; Sharma et al., 2021; Uprety et al., 2011). The study site is home to 20 mammalian species, including large carnivores such as leopard (DFO, 2019). The climate of the study area are sub‐tropical and tropical, with a mean annual rainfall of 1761 mm, a maximum temperature between 38°C and 41°C, and minimum temperature between 5°C and 60°C, whereas rainfall distribution is monsoon‐type between June and September (Sharma et al., 2021; Uprety et al., 2011).

### 2.2 Scat identification and collection

We conducted our survey between August and December 2020 in habitats including forests, grasslands, riverbeds, and roads in order to collect a sufficient number of scats. An average of 1.8 km transect walk was conducted within 36 grids, covering a total of 64.8 km within the study area. All together, we collected 96 leopard scats. The scats of the leopards were identified based on their shape, size, and pugmark. The leopards' scats were much larger than those of other small cat species such as jungle cats and hyenas. The pugmarks and scratch mark of the leopard were supplementary evidence of leopard scat. Moreover, the size and appearance of leopards's scats more or less overlap with those of other large Falidae, such as tigers (*Panthera tigris*). Due to the absence of tigers at this study site, it was easy to identify and confirm the leopard scats. However, unidentified scats were discarded from the collection. The date of collection and GPS location were recorded for each sample before it was stored in zip‐locked bags for further analysis.

### 2.3 Laboratory analysis

All scats were washed with cold water to remove the soil and leaves. If there were undigested items in the scat, such as bones, they were also removed. They were then sun‐dried for 24 h. The dried samples were labeled and stored in the paper bags for treatment. Following the method of Bhandari et al. (2020), we prepared slides imprinting the cuticle pattern of sample hairs of prey species found in leopard scats. A total of 20 hairs were picked randomly from each sample and soaked in a 1:1 alcohol and diethyl ether solution for 30 min. The hairs were then dried at room temperature. From these, five hairs were randomly selected and laid out in parallel lines on a slide that was painted with transparent nail polish to observe the cuticle pattern of the hairs. These hairs were then removed from the slide, and the imprint of the hair on the slide was observed through a compound stereoscopic microscope at 400× magnification.

The recorded cuticle images of the hairs extracted from the scat were then compared using a reference key for species confirmation. The reference images required for the comparison were also prepared following the aforementioned method. The hair sample and mean live weight of the prey species and small mammals and carnivores were obtained from the Pokhara Zoological Park, Kaski, operated by the Division Forest Office, Kaski, Nepal. We used the regression equations developed by Floyd et al. (1978) and Ackerman et al. (1984) to estimate the relative proportion of biomass of different prey species consumed by the leopard. The regression equations relate the average live weight of a prey animal consumed (*X*) to the weight of consumed prey represented by one field‐collectible scat (*Y*):

Y=1.98+0.035x.

The term *Y* is the biomass of prey consumed (kg) to produce a single field collectable scat, and *X* is the average body weight of the prey species (in kg). Relative biomass kill (*D*) and relative number of individual kill (*E*) were calculated. Where (*A*) is the occurrence, and (*B*) is the live weight of the prey species. We followed published literature such as Link and Karanth (1994), Karanth and Sunquist (1995), and Biswas and Sankar (2002) for the calculations.

$$D=\frac{\left(A\times C\right)}{\sum \left(A\times C\right)}\;\text{and}\;E=\frac{\left(D÷B\right)}{\sum \left(D÷B\right)}.$$

We also used Evlev's electivity index (*E*_*i*_) to measure the relationship between the proportion of prey species found in the cats and the prey available in nature.

$$E_{i}=\frac{\left(r_{i}-p_{i}\right)}{\left(r_{i}+p_{i}\right)},$$

where *r*_*i*_ represents the relative abundance of a prey in a leopard's diet and *p*_*i*_ is the prey density. In this equation, *E*_*i*_ = ranges from −1 (total avoidance) to 1 (high preference). To know the prey status, prey abundance data was obtained from a camera trap survey. The camera traps were deployed for a total of 21 nights at 36 locations. The photographic captures with >30 min interval were regarded as independent captures. To compute the abundance for each species, the total independent captures for each species were summed for all camera traps over all days and divided by the total number of camera trap days.

## 3 RESULTS

**FIGURE 2:**
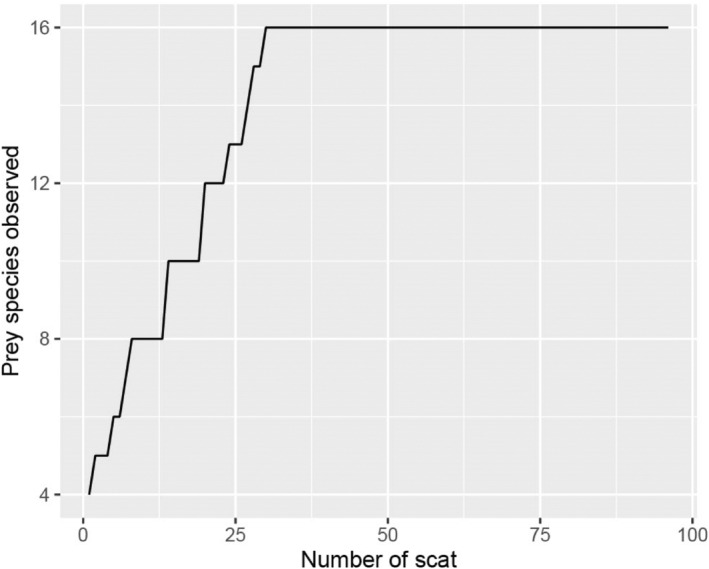
Total number of scat = 96 is represented in *X* axis, whereas the number of species observed is represented in *Y* axis. The number of prey species reached the asymptote at the sample size of 31.

**FIGURE 3:**
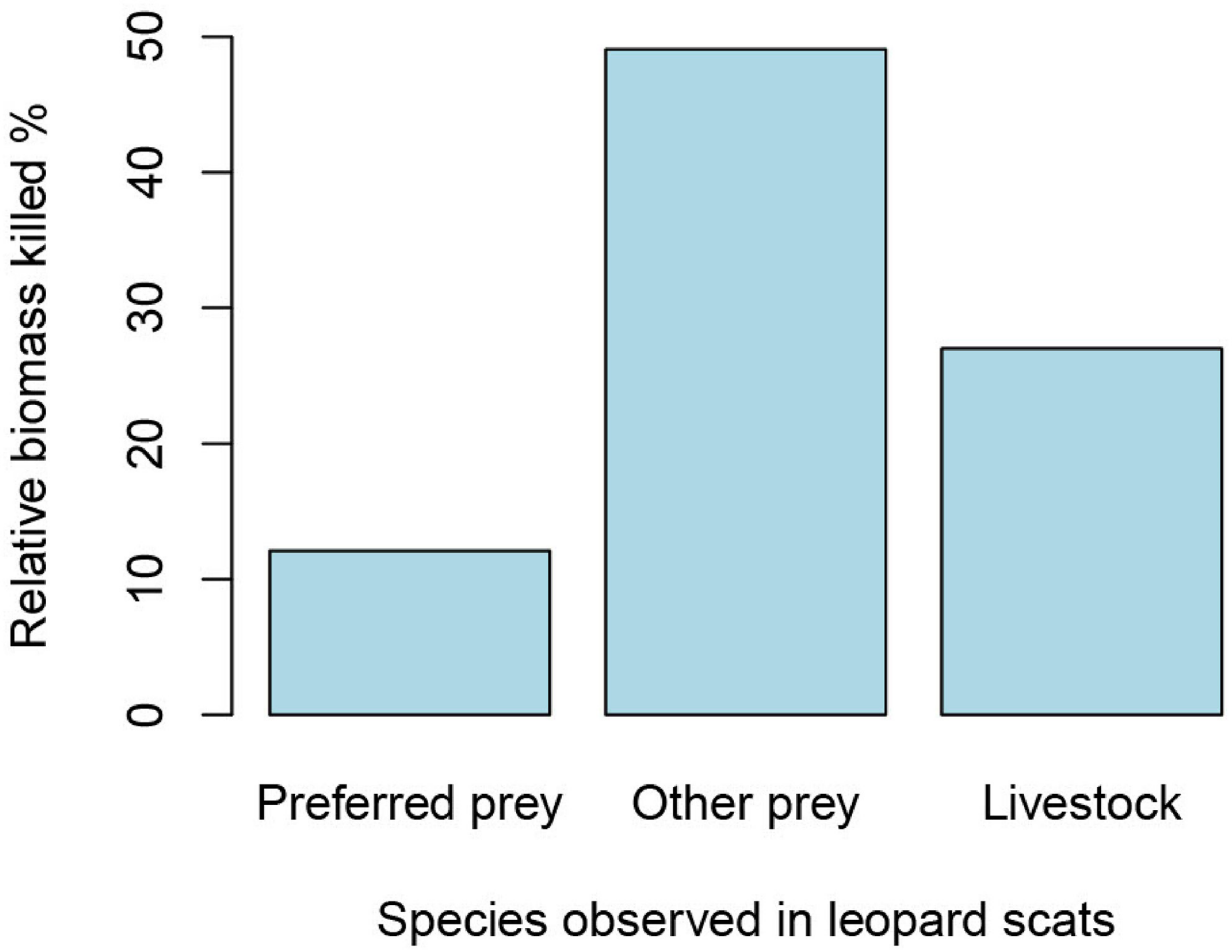
The percentage of relative biomass killed among preferred prey, other prey, and livestock was observed in leopard scats.

We observed a total of 15 prey species in 96 leopard scats (Table 1). Plastic, bird feathers, and unidentified items were also found in four scats. The number of prey species reached an asymptote at a sample size of 31 (Figure 2). Overall, goats contributed the highest relative biomass among the killed species, followed by jungle cats, domestic dogs, and large Indian civets (Table 1). A total of 27% of the relative biomass killed came from livestock (Figure 3), with goats contributing the most relative biomass among the livestock found in leopard scats (Table 1). Among the wild prey, the relative biomass killed for wild ungulates (barking deer and wild boar) was only 10%, while the relative biomass killed for other wild prey was approximately 50% (Figure 3). Jungle cats (12%) and large Indian civets (10%) had the highest relative biomass killed among wild prey, compared to other carnivores, making them more or less preferred prey for leopards. Similarly, wild ungulates had the lowest relative number of individuals killed (~3%). However, we observed a higher relative number of individuals killed for some other species, such as (16.2%) for the Indian hare, (10.2%) for the Jungle cat, and (9.2%) for the large Indian civet. Moreover, the relative number of individuals killed for goats and domestic dogs was (4.3%) and (5.8%), respectively (Table 1).

**TABLE 1:** A summary of the scat analysis of leopards in the mid‐hills of Nepal, with a total number of scats = 96, where RBK = relative biomass killed and RIK = relative number of individuals killed.

Species	Composition	(*A*) Occurrence %	(*B*) Live weight “*X*” (kg)	(*C*) Correction factor *Y*	(*D*) RBK (%)	(*E*) RIK (%)
Goat (*Capra hircus*)	18	8.4	20	9.0	14.3	4.3
Wild boar (*Sus scrofa*)	3	1.4	38	15.3	4.1	0.6
Barking deer (*Muntiacus muntjak*)	8	3.7	18	8.3	5.9	2.0
Domestic dog (*Canis familiaris*)	21	9.8	12	6.2	11.5	5.8
Large Indian civet (*Viverra zibetha*)	27	12.6	7	4.4	10.6	9.2
Masked palm civet (*Paguma larvata*)	6	2.8	4	3.4	1.8	2.7
Indian hare (*Lepus nigricollis*)	18	8.4	1.5	2.5	4.0	16.2
Crab eating mongoose (*Herpestes urva*)	12	5.6	2	2.7	2.8	8.6
Yellow throated marten (*Martes flavigula*)	9	4.2	3.5	3.2	2.6	4.4
Rhesus monkey (*Macaca mulatta*)	6	2.8	8	4.8	2.5	1.9
Domestic cat (*Felis catus*)	6	2.8	2.5	2.9	1.5	3.7
Leopard cat (*Prionailurus bengalensis*)	12	5.6	2.5	2.9	3.0	7.4
Jungle cat (*Felis chaus*)	30	14.0	7	4.4	11.8	10.2
Golden jackal (*Canis aureus*)	12	5.6	11	5.8	6.2	3.4
Indian crested porcupine (*Hystrix indica*)	12	5.6	7	4.4	4.7	4.1
Birds	5	2.3	0	0	0	0
Plastic	3	1.4	0	0	0	0
Unknown/grass	4	1.9	0	0	0	0
Total	215	100				

**FIGURE 4:**
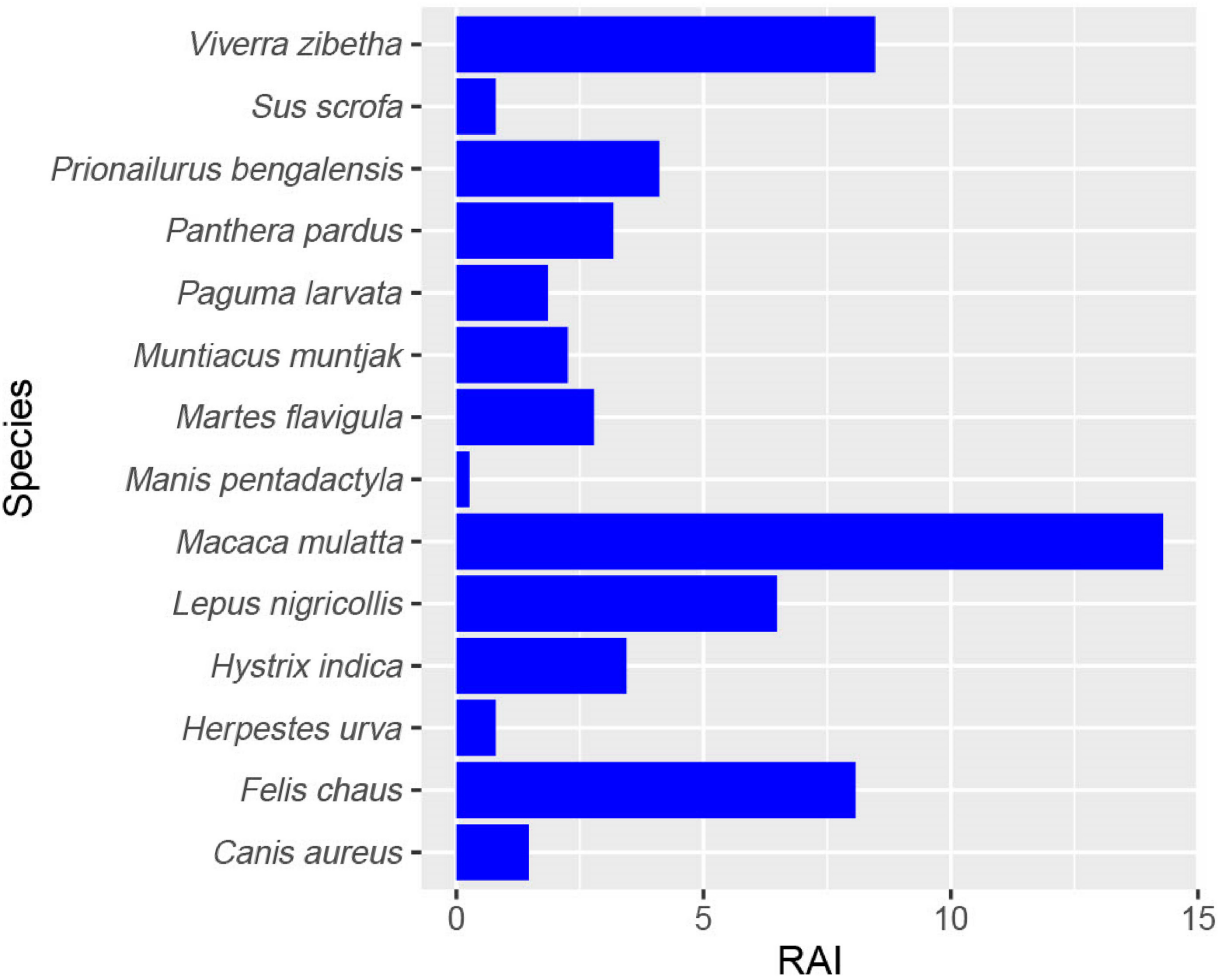
Relative abundance of prey species and leopard generated from camera trap survey in the Bhanu Municapility between October and December 2020.

**FIGURE 5:**
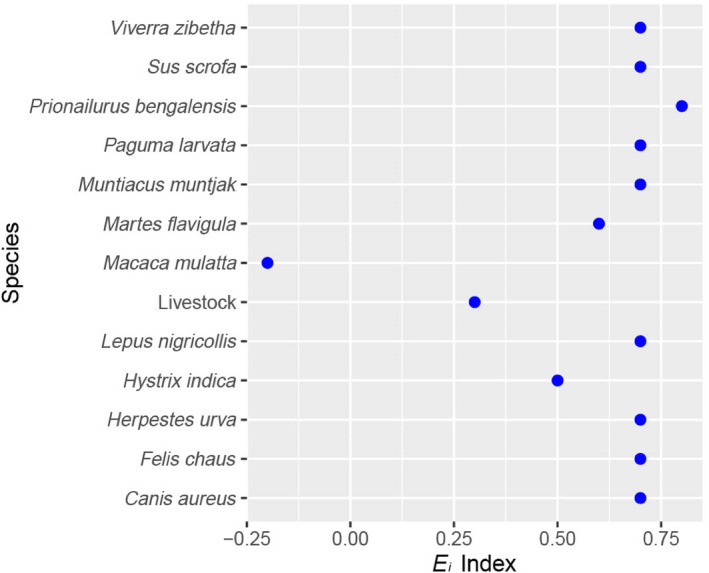
Evlev's selectivity index of the prey species based on leopard scat analysis. All species were positive selected except rhesus monkey.

The findings obtained from the camera trap showed that the relative abundance of wild ungulates (barking deer and wild boar) was notably low, while rhesus monkeys were the most abundant, followed by large Indian civets, jungle cats, and Indian hares (Figure 4). We observed that the Ivlev's electivity index indicated that except for rhesus monkeys, all the species were preferred for the leopard (Figure 5). The leopard cat was one of the most preferred species for the leopards' prey (Figure 5). We found that the majority of the scats (49%) were found on wide roads (>4 m in breadth), followed by narrow roads (1–4 m) (28%) and trails (~1 m) (19%). Scats were found in mixed forests (34%), sal‐dominated forests (31%), shrubs (23%), and others (grassland and agriculture land) (12%).

## 4 DISCUSSION

In our study, we found 15 different prey species in the leopard's diet, surpassing the findings of previous studies (Lovari et al., 2015; Thapa, 2011). Hayward et al. (2006) suggested that leopards have one of the most diverse diets among carnivores. Unlike a previous study (Mukherjee et al., 1994), where 48% of scat samples contained a single prey species, none of the scats in our study consisted of just one prey species. This greater prey diversity in our scat samples may be attributed to the absence of large ungulates like chital and sambar in our study area, which potentially led leopards to hunt a variety of smaller prey, resulting in increased prey diversity in their scats.

### 4.1 Relative biomass of wild and domestic prey

In the leopard scat analysis, small carnivores like the jungle cat and large Indian civet made up the majority of the relative biomass, even though they are not typically a significant part of the leopard's diet (Kandel et al., 2020; Lovari et al., 2015; Thapa et al., 2014). Conventionally preferred prey species for leopards in Nepal's mountain region, such as wild boar and barking deer, only accounted for 10% of the total relative biomass in this study. This shift may be due to the scarcity of ungulates like barking deer and wild boar in the study area, pushing leopards to rely more on smaller wild mammals that were more abundant.

In our study, domestic prey made up 27% of the relative biomass, with goats (~15%) and dogs (~12%) being the most significant contributors to the leopard's diet. Leopards exhibit a flexible hunting strategy that allows them to adapt to human‐dominated landscapes, as noted by Thapa (2011), who observed their ability to modify their foraging and dietary habits (Ramesh et al., 2012). These human‐dominated areas often have an abundance of domestic prey, including goats, sheep, dogs, cows, buffaloes, and poultry, which serve as a substitute diet for leopards. In regions with high human activity, domestic livestock have been found to make up a substantial part of the leopard's diet (Athreya et al., 2013; Khorozyan et al., 2015; Kumaraguru et al., 2011). For instance, Athreya et al. (2015) reported that in a heavily urbanized area in India, majority of the leopard's prey biomass consisted of domesticated animals. However, in our study area, the proportion of domestic animals in the leopard's diet is lower. This difference could be attributed to the fact that the Indian study took place in a highly urbanized, anthropogenic setting, while our study occurred in a semi‐urban area with medium to high levels of human disturbance.

### 4.2 Relative individual killed

Small mammals, such as the Indian hare and jungle cat, constituted the largest proportion of individually killed prey and are frequently preyed upon by leopards compared to other prey. This may be due to their high abundance and small body size. Since leopards need to consume smaller‐bodied individuals to generate the same amount of energy compared to preying on larger species, which provide equal energy even with fewer individuals killed. Numerous studies have reported instances of leopards preying on other smaller carnivores (Baral, Bhandari, & Adhikari, 2022; Hayward et al., 2006; Thapa, 2011). The preference for specific individuals is believed to be the major factor governing leopard behavior when preying on other carnivores (Baral, Bhandari, & Adhikari, 2022; Hayward et al., 2006). However, the exact reason for this behavior remains unclear and requires further detailed study.

### 4.3 Influence of body weight on the prey preference

Prey size is a crucial factor influencing the leopard's choice of prey, as observed in previous studies. These studies have noted the leopard's preference for medium‐sized prey species such as chital, wild boar, and hog deer (Bhattarai & Kindlmann, 2012; Hayward et al., 2006; Lovari et al., 2015; Thapa, 2011). Moreover, other research has shown that the leopard's broad diet falls within the preferred prey body weight range of 10–40 kg (Andheria et al., 2007; Hayward et al., 2006; Karanth & Sunquist, 1995; Ramesh et al., 2009).

Livestock, such as goats and dogs, typically weigh more than 10 kg and are comparable in size to the leopard's conventionally preferred prey, like barking deer and wild boar. This similarity in size may explain the leopard's inclination to target domestic animals as prey. Carnivores seek to minimize the time and energy expended in hunting, as it requires substantial energy to capture prey. Pursuing and subduing larger prey, in particular, results in a twofold increase in energy expenditure. Consequently, leopards may focus their hunting efforts on unguarded livestock near human settlements because they are easier to capture than wild ungulates (Khan et al., 2018; Penjor et al., 2022). Leopards, well‐suited for medium‐sized prey, may resort to smaller prey during tough times when preferred prey is scarce (Hayward et al., 2006; Karanth & Sunquist, 1995). However, the role of alternative prey in their diet is poorly understood. In areas where their preferred prey is scarce and energy‐intensive to hunt, leopards may opt for smaller mammals and domestic animals. Unlike lions, which can tackle larger prey in groups, leopards' solitary nature may restrict them from capturing large domestic animals like buffalo and cows. This could explain why we found no instances of cows and buffalo in the leopard diet but observed a notable presence of smaller livestock like goats and dogs among domestic prey.

### 4.4 Ivlev electivity index

Rhesus monkeys are highly abundant in the study area, but they make up a relatively small proportion of the biomass killed by leopards. Interestingly, leopards also tend to avoid preying on this species. The rhesus monkeys' arboreal nature and their group vigilance likely play a role in protecting them from predators like leopards. Prior studies have similarly noted that leopards typically turn to primates as prey only when other prey options are limited (Hayward et al., 2006). Despite their abundance, this is likely one of the reasons why monkeys were the only species with a negative value in Ivlev's electivity index. On the other hand, the large Indian civet emerged as one of the highly preferred species, possibly due to its high abundance in the leopard's habitat.

### 4.5 Relation of leopard's diet with human leopard conflict

In areas like our study site, where natural prey is scarce, leopards may increasingly turn to domestic livestock and even humans as alternative prey sources (Adhikari, Baral, Bhandari, Kunwar, et al., 2022; Rostro‐García et al., 2016). The human‐leopard conflict is a growing concern, primarily driven by leopards shifting from wild prey to domestic options (Athreya et al., 2015). With smaller wild prey insufficient to meet their energy needs, leopards are likely to hunt in areas with human settlements and agriculture, especially in regions with high human activity and limited natural prey. This, in turn, leads to increased conflicts (Athreya et al., 2015; Khorozyan et al., 2015). To foster coexistence between humans and leopards, it is crucial to reduce negative interactions, ideally by implementing sustainable conservation strategies for wild prey such as barking deer and wild boar.

## 5 CONCLUSION

Small carnivores like jungle cats and large Indian civets, along with domestic animals such as goats and dogs, make up the primary components of the leopard diet in Nepal's mid‐hill region. This heavy reliance on small mammals and livestock is likely to lead to increased negative interactions between humans and carnivores (Bhandari & Chalise, 2016). Additionally, the significant consumption of livestock and small mammals by leopards suggests a scarcity of medium‐sized wild prey, particularly preferred ungulate species like barking deer and wild boar. To ensure effective leopard conservation, it is crucial to implement management strategies such as habitat restoration techniques for conserving wild ungulates. Similarly, raising awareness and establishing wildlife relief or compensation programs can reduce conflicts between humans and leopards, fostering coexistence in these areas.

## AUTHOR CONTRIBUTIONS

**Kedar Baral:** Conceptualization (lead); data curation (equal); formal analysis (equal); funding acquisition (equal); investigation (lead); methodology (equal); project administration (lead); software (equal); visualization (lead); writing – original draft (lead); writing – review and editing (lead). **Shivish Bhandari:** Data curation (equal); formal analysis (equal); writing – review and editing (equal). **Binaya Adhikari:** Data curation (equal); formal analysis (equal); writing – review and editing (equal). **Ripu M. Kunwar:** Supervision (equal); writing – review and editing (equal). **Hari P. Sharma:** Supervision (equal); writing – review and editing (equal). **Achyut Aryal:** Supervision (equal); writing – review and editing (equal). **Weihong Ji:** Supervision (lead); writing – review and editing (equal).

## CONFLICT OF INTEREST STATEMENT

None declared.

## Data Availability

The dataset that is associated with this study is available at dryad https://doi.org/10.5061/dryad.x95x69prf.
